# Metformin Ameliorates Podocyte Damage by Restoring Renal Tissue Podocalyxin Expression in Type 2 Diabetic Rats

**DOI:** 10.1155/2015/231825

**Published:** 2015-05-13

**Authors:** Limin Zhai, Junfei Gu, Di Yang, Wei Wang, Shandong Ye

**Affiliations:** Department of Endocrinology, Anhui Provincial Hospital Affiliated to Anhui Medical University, Hefei 230001, China

## Abstract

Podocalyxin (PCX) is a signature molecule of the glomerular podocyte and of maintaining integrity of filtration function of glomerulus. The aim of this study was to observe the effect of different doses of metformin on renal tissue PCX expression in type 2 diabetic rats and clarify its protection on glomerular podocytes. Type 2 diabetic Sprague-Dawley (SD) rats in which diabetes was induced by high-fat diet/streptozotocin (HFD-STZ) were treated with different doses of metformin (150, 300, and 500 mg/kg per day, resp.) for 8 weeks. Various biochemical parameters, kidney histopathology, and renal tissue PCX expression levels were examined. In type 2 diabetic rats, severe hyperglycemia and hyperlipidemia were developed. Urinary albumin and PCX were markedly increased. Diabetes induced significant alterations in renal glomerular structure. In addition, protein and mRNA expression of renal tissue PCX were highly decreased. However, treatment of rats with different doses of metformin restored all these changes to a varying degree. These results suggested that metformin can ameliorate glomerular podocyte damage in type 2 diabetic rats, which may be partly associated with its role in restoring PCX expression and inhibiting urinary excretion of PCX with dose dependence.

## 1. Introduction

Type 2 diabetes is becoming an emergent issue in the world. Diabetic nephropathy (DN) is one of the most serious microvascular complications of diabetes, which is the leading cause of end-stage renal disease [1]. Previous studies have reported that podocytes are highly differentiated, insulin-dependent glomerular epithelial cells contributing to glomerular filtration barrier [2]. There are several proteins on podocyte membrane that are important in the regulation of the glomerular filtration such as nephrin, podocin, and podocalyxin [3, 4]. Podocalyxin is a sialoprotein contributing to the negative charge of glomerular membrane and maintaining integrity of filtration function of the glomerulus [5]. Recently, some researches reported that metformin (1,1-dimethylbiguanide), as the first-line hypoglycemic drugs for the treatment of T2DM, can prevent and delay the occurrence and progress of diabetic nephropathy through some mechanisms, which include improving glucolipid metabolism, improving insulin resistance, and some other nonhypoglycemic action mechanisms [6]. In this study, we aim to investigate the protective effect and possible mechanisms of metformin on podocyte damage in type 2 diabetic rats so as to provide an experimental basic for the use of metformin in the clinical prevention and treatment of DN.

## 2. Materials and Methods

### 2.1. Animals

Forty-five male Sprague-Dawley (SD) rats (180–200 g) aged two months were purchased from the Experimental Animal Center of Anhui Medical University. Each three rats were fed in one cage with enough food and water under condition of relative humidity of 40%–60% and temperature of 20–30°C. Ventilation and lighting were supported regularly. During the experiment period, animals were treated strictly according to the rules in the Ethics Committee of Anhui Provincial Hospital Affiliated to Anhui Medical University. Every effort was made to minimize the number of animals used and their suffering.

### 2.2. Reagents and Apparatuses

The following reagents were used: STZ (Sigma, USA), metformin (Shanghai Shiguibao Medicine Co., Ltd., China), TC, TG, and LDL-C kits (Beijing BHKT Clinical Reagent Co., Ltd.), urine creatinine (Ucr) and urea nitrogen (BUN) kits (Jiancheng Technology Co., Nanjing, China), urinary albumin (Ualb) kit (Tianjing, Xiehe Medicine Co., Ltd., China), urinary PCX ELISA test kit (Shanghai Dr. Dean Biotechnology Co., China), IHC kits (Abcam, France), and TRIzol, real-time PCR kit, and primers of PCX (TaKaRa, China).

Apparatuses Ultra 2 full-automatic glycosylated hemoglobin analyzer (Primus, USA), SN-695B *γ*-counter (China), HT1 enzyme-linked immune detector (Biocell, Austria), UV-2600 UV-Vis spectrophotometer (Youni Ke Instrument Co., Ltd., Shanghai), JEM-1230 transmission electron microscopy (JEOL, Japan), and an ABI7500 apparatus (Bio-Rad, USA) were used in this study.

### 2.3. Type 2 Diabetic Rats Modeling

T2DM was induced by high-fat diet and low-dose STZ as mentioned previously [7–9]. Briefly, SD rats were fed a high-fat diet (a basal diet + 10% lard compound + 2% cholesterol, 50% calories from fat) for 4 weeks followed by single intraperitoneal (i.p.) injection of a low dose of streptozotocin (STZ, 30 mg/kg, in citrate buffer, pH 4.5). After 72 hours, blood glucose level was measured. Modeling was considered to be successful if blood glucose level was higher than 16.7 mmol/L accompanied with dieresis and polydipsia. These rats were fed a high-fat diet throughout the whole study.

Methods: 8 SD rats served as normal control group (group NC, *n* = 8) and sterile citrate buffer (i.p.) was administered to them. T2DM rats were divided randomly into group DM (*n* = 9) treated with vehicle alone or group M1 (*n* = 8), group M2 (*n* = 8), or group M3 (*n* = 7) treated with metformin (150, 300, and 500 mg/kg per day, resp.) for 8 weeks. The dose of metformin was based on studies with long-term metformin treatment in STZ-diabetic rats [10]. Different doses of metformin were intragastrically administered after being milled.

### 2.4. Biochemical Detections

24 h urinary samples were collected at the end of 8 weeks and then centrifuged at 2,000 g for 10 min for the determination of urinary albumin (Ualb), urinary sediment PCX (Upcx), and urinary creatinine (Ucr). Ualb was measured by radioimmunoassay. Upcx was measured by enzyme-linked immunosorbent assay. Jaffe's assay was used to measure Ucr. To eliminate the impact of urine volume, urinary albumin and urinary sediment PCX were expressed as urinary Ualb/Ucr (UACR) and Upcx/Ucr (UPCR), respectively. These were calculated using the following equations:

 UACR (mg/g) = Ualb (mg/L) ÷ [Ucr (*μ*mol/L) × the molecular weight of Cr (113.12 g/mol) × 10^−6^].
 UPCR (ng/g) = Upcx (ng/L) ÷ [Ucr (*μ*mol/L) × the molecular weight of Cr (113.12 g/mol) × 10^−6^].

Then all rats were anesthetized and blood was withdrawn from abdominal aorta for the detection of HbA1c, insulin (INS), serum BUN, TG, TC, and LDL-C. HbA1c was tested by using high-performance liquid chromatography (HPLC). Insulin was tested by the radioimmunoassay method. BUN was tested by the urease method, TG and TC were tested by the enzyme reaction, and LDL-C was tested by selective precipitation method.

### 2.5. Histopathology Observation

All rats were sacrificed; then the pathological changes of the kidney tissue were observed by electron microscope. 1 mm^3^ renal cortex was fixed in 2.5% glutaraldehyde to produce ultrathin section. Ultrathin sections were observed by electron microscopy at magnification of 10,000. Two glomeruli were observed for each sample. Ten photos were taken from each glomerulus through continuous manual acquisition. Average harmonic mean of GBM in each group was calculated as GBMT of the corresponding group [11, 12].

From each photo, the GBM and the part of fused foot process were traced and measured in an image processing and analysis program (Image Pro Plus 6.0). Foot process fusion rate (FRFP) = the total length of GBM/the sum length of fused foot process covered in the corresponding GBM. For each photo, FRFP was calculated and used to finally calculate a mean FRFP for each group [13, 14].

### 2.6. Renal Tissue PCX Protein Expression

Small blocks of kidney tissue were embedded in paraffin, following dewaxing and adding antigen for microwave repair. Endogenous peroxidase was quenched by 3% hydrogen peroxide for 10 min. Small blocks were blocked by 5% BAS for 10 min. Kidney sections were stained for PCX (1 : 300) at 37°C for 3 h. Then polymerization-HRP anti-rabbit IgG was used for incubating sections at 37°C for 30 min. The renal PCX protein expression was employed by integrated optical density (IOD) detection. Image Pro Plus 6.0 was used to calculate the average IOD of the positive reaction of 10 images of each sample. The sections were examined with light microscopy by an experienced pathologist, and 10 images of each sample were acquired by continuous manual acquisition with magnification of 400.

### 2.7. Renal Tissue PCX mRNA Expression

Total RNA was isolated using TRIzol and reverse-transcribed using a reverse transcription kit. Real-time PCR was performed on an ABI7500 apparatus using SYBR green. Duplicate PCR reactions were tested using the following amplification protocol: 95°C for 10 seconds followed by 40 cycles at 95°C for 5 seconds and at 60°C for 34 seconds. Upstream and downstream primers of PCX gene sequence were CCA CAG CCC TAC CAA CCA and GTG TGT GAA TTC TTC GGT CGT. Upstream and downstream primers of *β*-actin gene sequence were GCC T TA GCC TGG ACC CA T AGT and GAC CAC CAA TCC ACA CAGA. Results were normalized to the *β*-actin mRNA levels and represented using the comparative threshold cycle method. The cycle threshold (Ct) value of each sample was analyzed by the 7500 software. The relative changes in gene expression were calculated using the relative quantitative method (2^−ΔΔCt^).

### 2.8. Statistical Analysis

All statistical analyses were performed using SPSS 16.0 (SPSS Inc., USA). The results were presented as means ± standard deviation (SD). For the data conforming to homogeneity of variances, one-way analysis of variance (ANOVA) was used to determine significant differences among groups, and Tukey's *b* test was used for comparison between individual groups. For the data not conforming to homogeneity of variances, Games-Howell test was adopted among groups. The results were considered significant when *P* < 0.05.

## 3. Results

### 3.1. Effect of Metformin on Weight and Indices of Serum Biochemical in Rats

**Figure 1:**
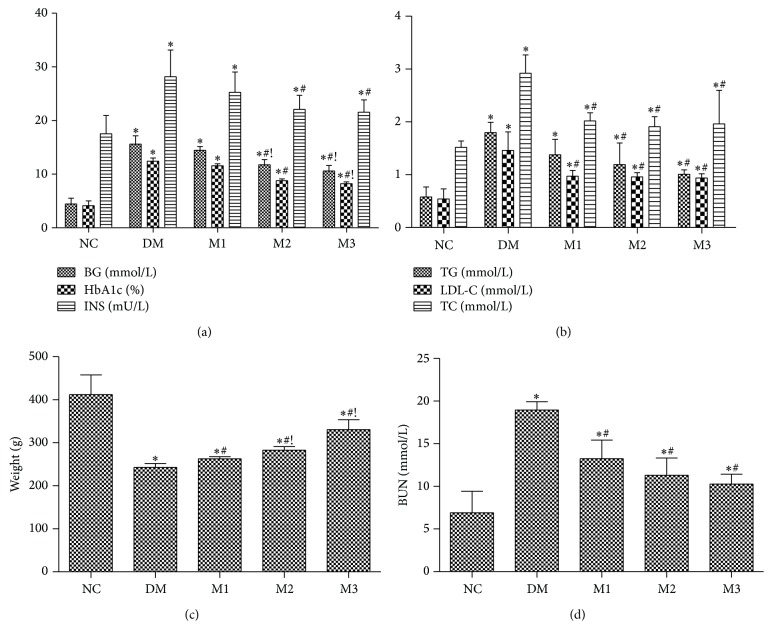
Effect of metformin on weight and indices of serum biochemical in rats. Different doses of metformin were administered to rats for 8 weeks. BG, HbA1c, and INS (a); TG, LDL-C, and TC (b); weight (c); and serum BUN (d) levels were determined as described in Materials and Methods. NC: normal rats; DM: HFD-STZ rats; M1: HFD-STZ + metformin (150 mg/kg per day) treatment rats; M2: HFD-STZ + metformin (300 mg/kg per day) treatment rats; M3: HFD-STZ + metformin (500 mg/kg per day) treatment rats. Data are expressed as mean ± SD. ^∗^ *P* < 0.05, versus NC group; ^#^ *P* < 0.05, versus DM group; ^!^ *P* < 0.05, versus M1 group. Games-Howell test was adopted to compare the level of weight and TC.

T2DM rats induced by high-fat diet/streptozotocin showed a profound elevation in the level of blood glucose, HbA1c, and INS (approximately 4-fold) while a marked decrease in the body weight as compared to NC group (Figures 1(a) and 1(c)) was noticed. In addition, induction of T2DM was evidenced by significant increases in TG, TC, LDL-C, and BUN after 8 weeks (Figures 1(b) and 1(d)). Treatment of T2DM rats with low dose of metformin (150 mg/kg) for 8 weeks only significantly decreased the levels of LDL-C, TC, and BUN (*P* < 0.05) but not BG, HbA1c, and INS, whereas higher doses of metformin (300, 500 mg/kg) obviously restored all measured biochemical parameters after 8 weeks (*P* < 0.05) as compared with group DM (Figure 1).

### 3.2. Effect of Metformin on Urinary Indices Levels in Rats

**Figure 2:**
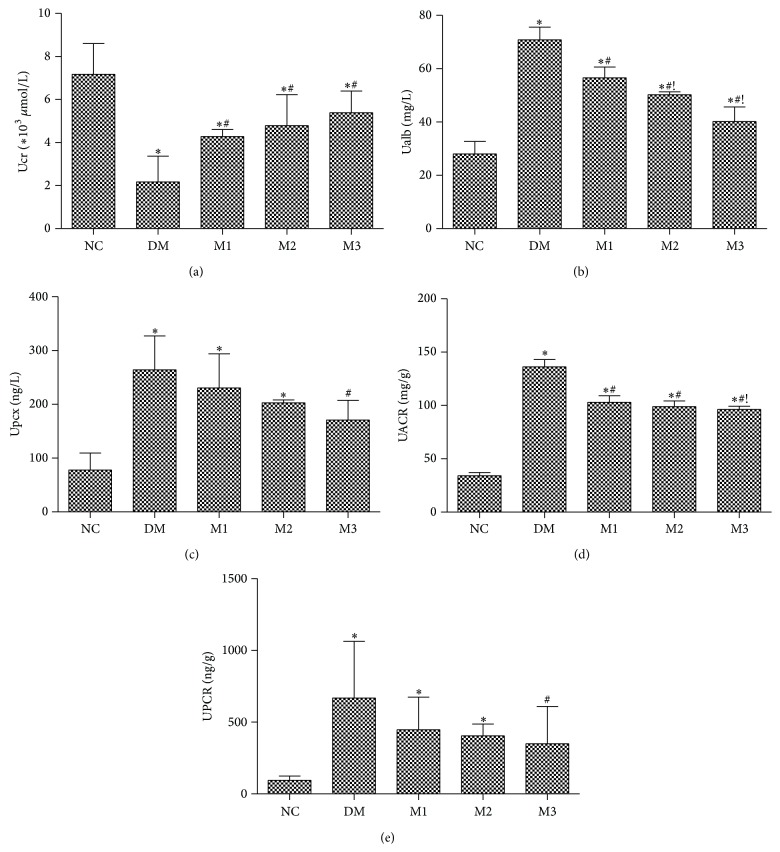
Effect of metformin on urinary indices levels in rats. Different doses of metformin were administered to rats for 8 weeks. Ucr (a), Ualb (b), Upcx (c), UACR (d), and UPCR (e) levels were determined as described in Materials and Methods. NC: normal rats; DM: HFD-STZ rats; M1: HFD-STZ + metformin (150 mg/kg per day) treatment rats; M2: HFD-STZ + metformin (300 mg/kg per day) treatment rats; M3: HFD-STZ + metformin (500 mg/kg per day) treatment rats. All values were expressed in mean ± SD. ^∗^ *P* < 0.05, versus NC group; ^#^ *P* < 0.05, versus DM group; ^!^ *P* < 0.05, versus M1 group.

Urinary sample collected from DM rats after 8 weeks showed a marked increase in Ualb, Upcx, UACR, and UPCR levels accompanied with a significant decrease in Ucr (Figure 2). Low dose of metformin treatment (150 mg/kg) caused a significant decrease in Ualb and UACR levels (*P* < 0.05); in addition, the highest dose of metformin (500 mg/kg) more noticeably caused decrease in Ualb and UACR as compared to group M1 (*P* < 0.05) (Figures 2(b) and 2(d)), while Figures 2(c) and 2(e) indicated only highest dose of metformin (500 mg/kg) significantly restored the increase in Upcx and UPCR (*P* < 0.05). On the other hand, metformin treatment obviously increased the level of Ucr as compared to group DM (*P* < 0.05) without difference between different MET groups (Figure 2(a)).

### 3.3. Effect of Metformin on the Renal Histopathological Changes in Rats

**Figure 3:**
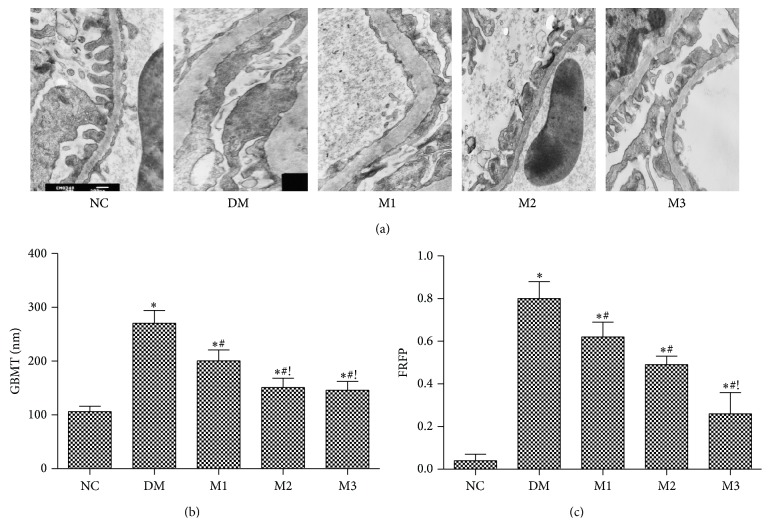
Effect of metformin on the renal histopathological changes in rats. The histopathological changes of renal tissue under electron microscopy among five groups (×10 000) (a); GBMT (b); FRFP (c). NC: normal rats; DM: HFD-STZ rats; M1: HFD-STZ + metformin (150 mg/kg per day) treatment rats; M2: HFD-STZ + metformin (300 mg/kg per day) treatment rats; M3: HFD-STZ + metformin (500 mg/kg per day) treatment rats. All values were expressed in mean ± SD. ^∗^ *P* < 0.05, versus NC group; ^#^ *P* < 0.05, versus DM group; ^!^ *P* < 0.05, versus M1 group.

Histopathological examination of kidney tissue obtained from NC rats showed normal GBM without foot process fusion. However, both the foot process fusion (nearly 80%) and GBM and GBMT (266.50 ± 10.23 nm) were found increased significantly in T2DM rats (*P* < 0.05); meanwhile, some foot processes were completely ruined and even vanished and the architecture of GBM became ambiguous (Figure 3). Both FRFP and GBMT were alleviated by MET treatment compared to those of group DM (*P* < 0.05). Compared to low dose of metformin (150 mg/kg) treatment, GBMT decreased much more (200.53 ± 20.36 versus 150.98 ± 17.25; 145.94 ± 16.10 nm) in higher doses of metformin (groups M2, M3) (*P* < 0.05) and FRFP was restored to 15% in M3 group (*P* < 0.05), as shown in Figure 3.

### 3.4. Effect of Metformin on the Renal Tissue PCX Protein and mRNA Expressions in Rats

**Figure 4:**
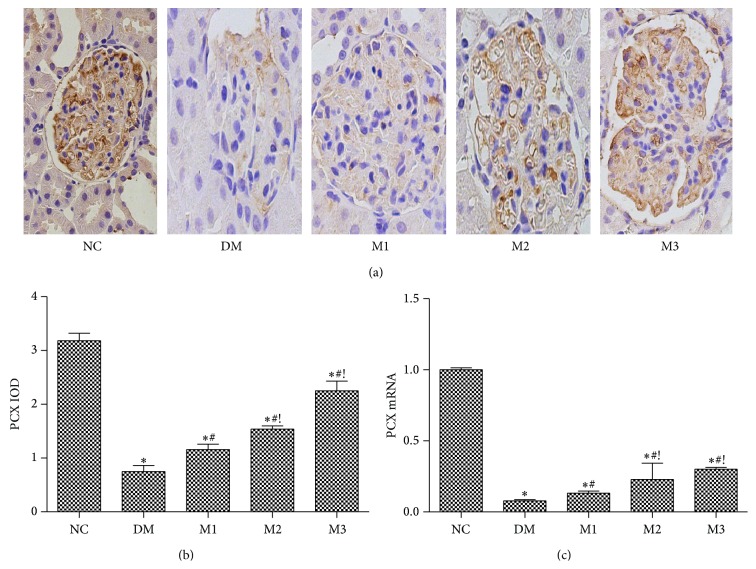
Effect of metformin on the renal tissue PCX protein and mRNA expressions in rats. (a) Immunohistochemical stating for PCX (×400); (b) the PCX IOD levels; (c) renal PCX mRNA level. NC: normal rats; DM: HFD-STZ rats; M1: HFD-STZ + metformin (150 mg/kg per day) treatment rats; M2: HFD-STZ + metformin (300 mg/kg per day) treatment rats; M3: HFD-STZ + metformin (500 mg/kg per day) treatment rats. Data are expressed as mean ± SD. ^∗^ *P* < 0.05, versus NC group; ^#^ *P* < 0.05, versus DM group; ^!^ *P* < 0.05, versus M1 group.

To investigate the role of metformin regulating the expression of PCX in renal tissue, the renal tissue PCX protein expression was detected within the visceral surface of Bowman's capsule in the podocyte with a membranous pattern of staining in normal control samples (Figure 4(a)). PCX protein expression decreased significantly in four diabetic groups which was accompanied with a significant decrease in PCX mRNA expression, especially in group DM (*P* < 0.05) when compared to the group NC (Figure 4). Treatment rats with different doses of metformin significantly restored the decreased protein and mRNA expression of PCX, which was more prominent in higher dose of metformin group (300, 500 mg/kg) than low dose of metformin group (150 mg/kg) (*P* < 0.05).

## 4. Discussion

Diabetic nephropathy is one of the most serious complications of both type 1 and type 2 diabetes mellitus [15] and also is one of the most common causes of end-stage renal disease. Metformin is recommended, in conjunction with lifestyle modification, as a first-line oral therapy in the recent guidelines of the ADA (American Diabetes Association) and EASD (European Association of the Study of Diabetes) [16, 17]. The main effect of this drug is to acutely decrease hepatic glucose production, mostly through mild and transient inhibition of the mitochondrial respiratory chain complex I [18]. Recently some experimental and clinical observations suggest that metformin has a specific protective effect on diabetic nephropathy (DN) independently of its hypoglycemic effect [10, 19]. In this study, the levels of serum BUN, UACR, and GBMT in T2DM rats were higher than those in group NC. These indexes were significantly decreased after MET treatment, the value of BG and HbA1c in M1 group had no significant changes compared with the group NC, while there was a significant decrease in serum BUN, UACR, and GBMT, which indicated that MET has a remarkable protective effect on the kidney independently of the hypoglycemic manner. The mechanisms are not clear completely, which may be related to its effects in activating AMP, activated protein kinase, improving oxidative stress, inhibiting inflammation and hyperfiltration of the kidney, reducing the formation of advanced glycation end products, lightening lipid deposition, and so forth [10, 19].

The filtration barrier of the kidney glomerulus that prevents plasma proteins from leaking into primary urine comprises endothelial cells, a glomerular basement membrane, and visceral epithelial cells (podocytes). Podocyte injury has been considered as one of the most important factors in diabetic nephropathy. Several medicines that protect from podocyte injury have a role in prevention of the development of diabetic nephropathy [20, 21]. In the present study, the foot cell fusion was gradually relieved by metformin treatment, especially in group M2 and group M3, which confirmed that MET can ameliorate podocyte damage in T2DM in a dose-dependent manner.

 Podocalyxin, located on the surface of podocytes that faces the urinary space, is one of the major negatively charged glomerular proteins in the glomerular basement membrane and plays an important role in the maintenance of the intricate glomerular basement membrane and plays an important role in the maintenance of the intricate glomerular podocyte for optimal filtration [22]. The increased urinary PCX (Upcx) excretion is a particularly sensitive index for indicating podocyte injury in patients with diabetes and can be used as an early specific indicator of glomerular podocyte dysfunction [23, 24]. In fact, decreased mRNA expression and protein components of slit diaphragm are related to the degree of proteinuria in several models of kidney diseases [25]. In our research, the Upcx was significantly increased in T2DM model rats compared to normal rats; moreover high dose of metformin (M3) remarkedly decreased Upcx excretion. PCX protein in normal rats was present along with glomerular peripheral capillary loops, which was reduced significantly in T2DM model rats. Treatment with high dose of metformin (M3) obviously inhibited the downregulation of PCX protein expression. Compared with group NC, the mRNA expression of PCX in group DM was obviously decreased, which was upregulated by MET treatment, and that was greater in groups M2 and M3 than in group M1 while the value of BG, HbA1c, and INS in M1 group had not been significantly restored compared with the group NC. From all the above, the effect of metformin adjusting renal tissue PCX expression is in a dose-dependent manner while it is independent of the hypoglycemic manner. Some researches elucidated possible underlying mechanisms of podocyte protection of metformin. Some researches elucidated possible underlying mechanisms: advanced glycation end products suppress PCX expression in high glucose-treated human podocytes through extracellular regulated protein kinases (ERK1/2) signal transduction pathway [26]. Hypoglycemic effect of metformin which was presented in this study can alleviate this suppression effect. Cholesterol is an important regulator in the development of proteinuric kidney disease and cholesterol homeostasis in podocytes is important to the formation of the slit diaphragm [27]. In the experiment, metformin decreased the level of TG, TC, and LDL-C, reduced the excretion of Ualb and Upcx, and relieved the FRFP. Therefore, we may infer that metformin develops its podocytes protection effect partly through ameliorating the level of lipid in DM rats. In addition to the contents discussed above, an experiment in cultured podocytes provided evidence that metformin can decrease ROS production through inhibiting NAD(P)H oxidase [28]. Kim et al. reported that metformin can suppress the diabetes-induced loss of podocytes through the repression of oxidative injury in a rat model of type 2 diabetes [29]. Similar mechanisms were proposed in which metformin can inhibit ecto-ATPase activity leading to an increased extracellular ATP concentration, subsequent activation of P2 receptors, and eventual reduction of NAD(P)H oxidase activity and enhancement of AMPK activity; these changes may have some beneficial effects for podocytes during metformin therapy [30]. The present study also presents the fact that a certain dose of metformin not only ameliorates hyperglycemia and hyperlipidemia, but also improves insulin level. These potential effects of metformin ameliorate podocyte damage together.

## 5. Conclusion

From all what is mentioned above, MET can reduce urinary PCX excretion as long as it adjusts the expression of PCX, so as to infer some podocyte protection in type 2 diabetic rats beyond its hypoglycemic effects. Specific mechanisms of this effect still need further researches.
